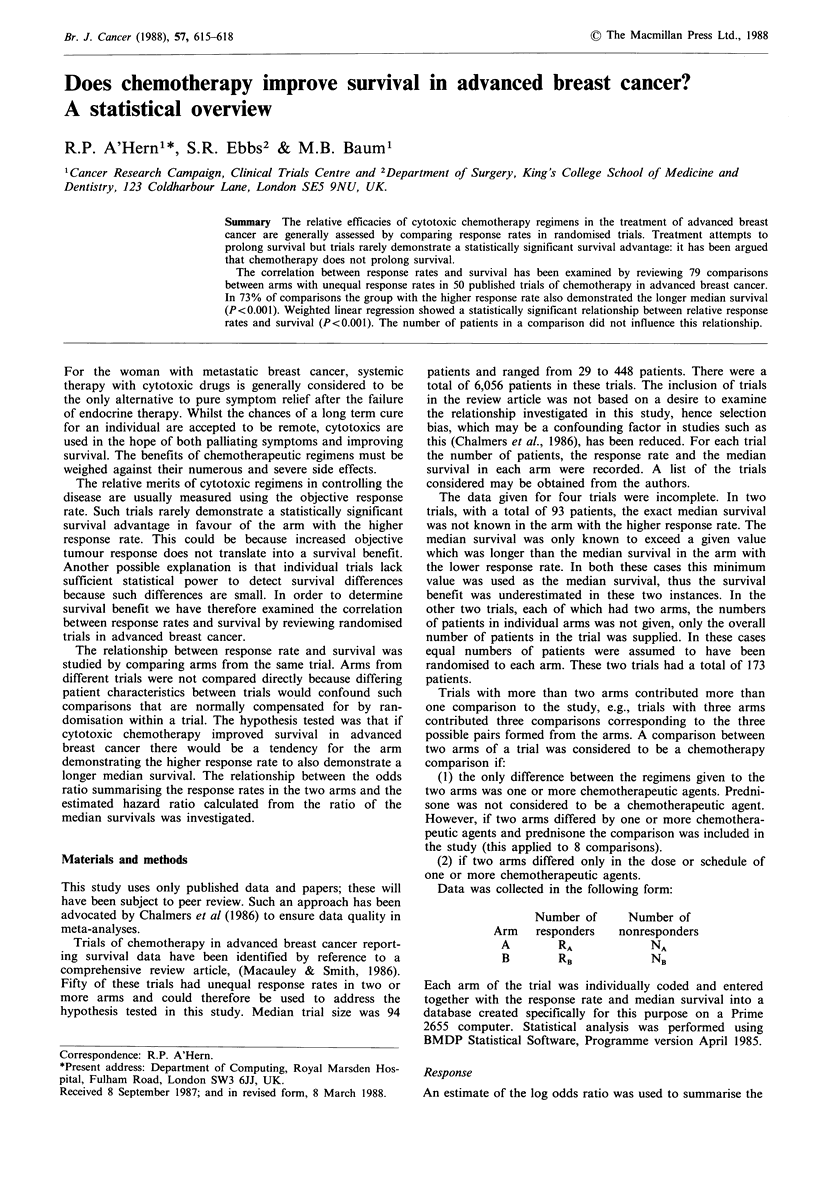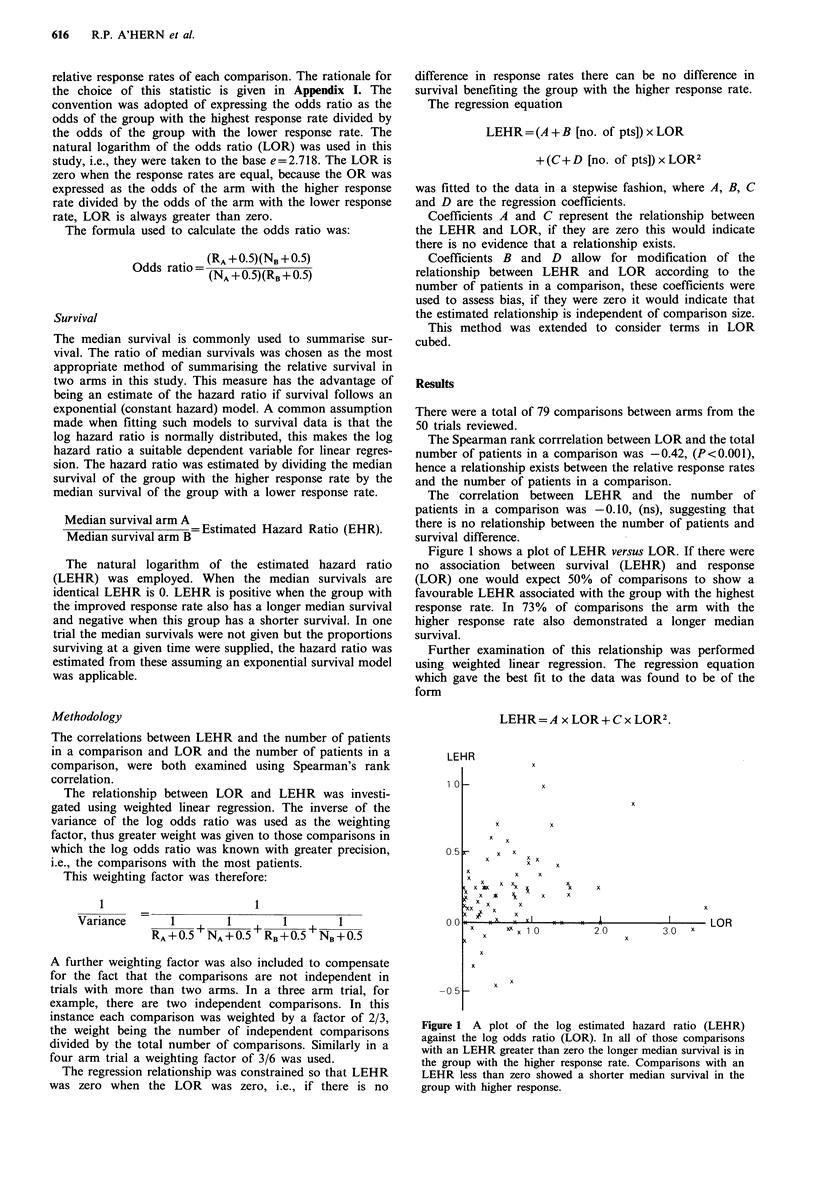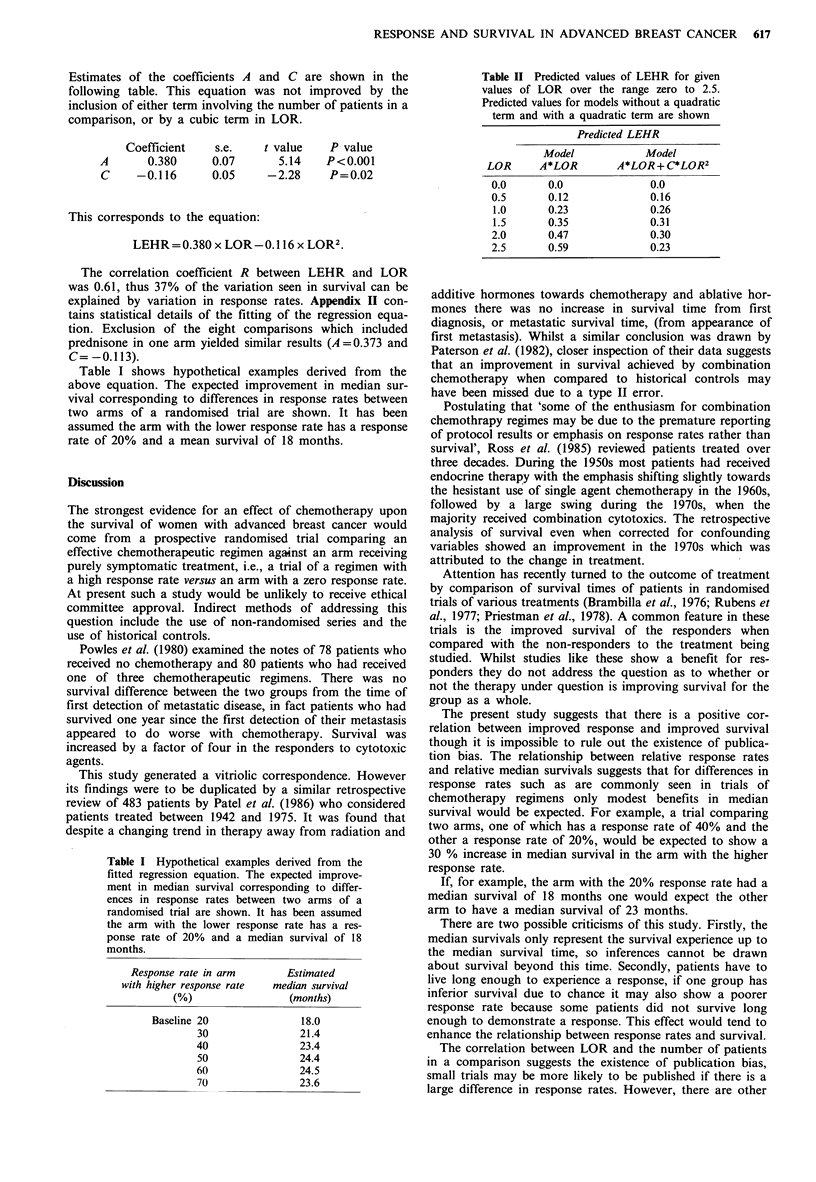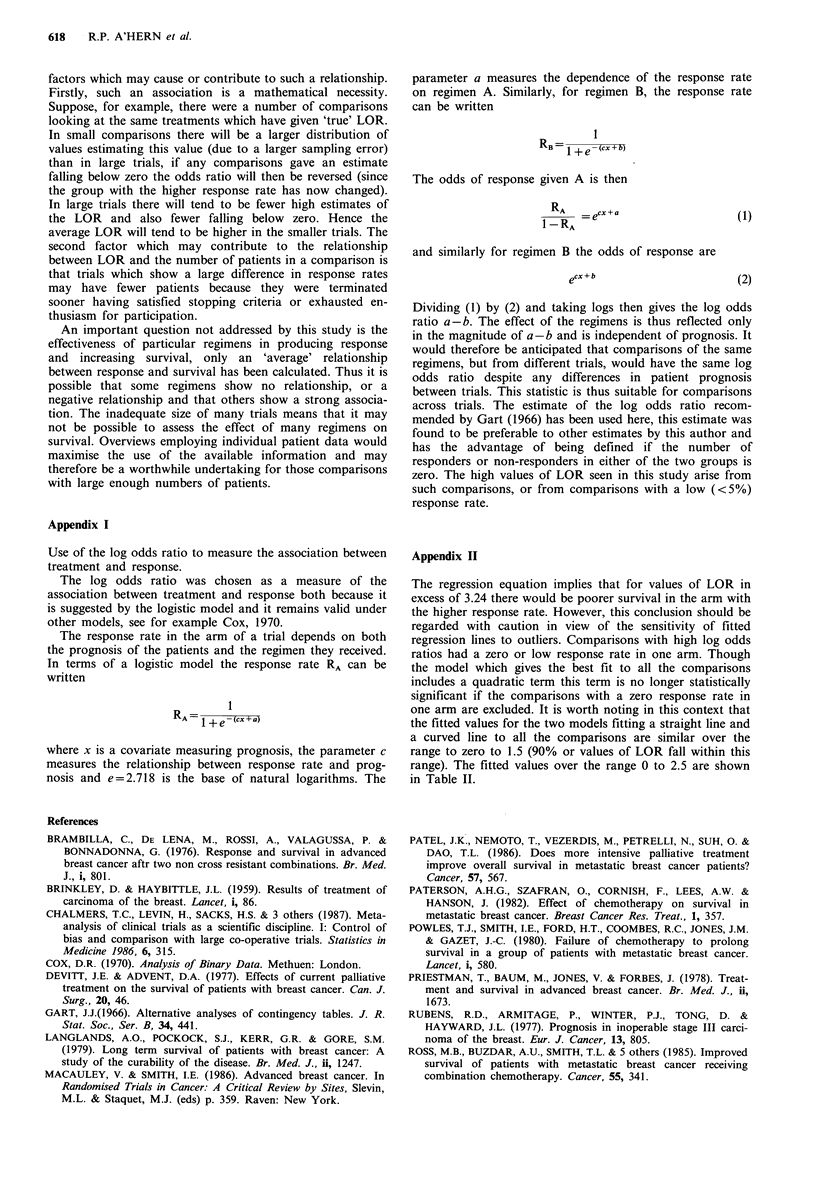# Does chemotherapy improve survival in advanced breast cancer? A statistical overview.

**DOI:** 10.1038/bjc.1988.140

**Published:** 1988-06

**Authors:** R. P. A'Hern, S. R. Ebbs, M. B. Baum

**Affiliations:** Cancer Research Campaign, Clinical Trials Centre, London, UK.

## Abstract

The relative efficacies of cytotoxic chemotherapy regimens in the treatment of advanced breast cancer are generally assessed by comparing response rates in randomised trials. Treatment attempts to prolong survival but trials rarely demonstrate a statistically significant survival advantage: it has been argued that chemotherapy does not prolong survival. The correlation between response rates and survival has been examined by reviewing 79 comparisons between arms with unequal response rates in 50 published trials of chemotherapy in advanced breast cancer. In 73% of comparisons the group with the higher response rate also demonstrated the longer median survival (P less than 0.001). Weighted linear regression showed a statistically significant relationship between relative response rates and survival (P less than 0.001). The number of patients in a comparison did not influence this relationship.


Br. J. Cancer (1988), 57, 615–618

© The Macmillan Press Ltd., 1988

### Does chemotherapy improve survival in advanced breast cancer? A statistical overview

R.P. A'Hern[1]*, S.R. Ebbs[2] & M.B. Baum[1]

[1]*Cancer Research Campaign, Clinical Trials Centre and* [2]*Department of Surgery, King's College School of Medicine and Dentistry, 123 Coldharbour Lane, London SE5 9NU, UK.*

**Summary** The relative efficacies of cytotoxic chemotherapy regimens in the treatment of advanced breast cancer are generally assessed by comparing response rates in randomised trials. Treatment attempts to prolong survival but trials rarely demonstrate a statistically significant survival advantage: it has been argued that chemotherapy does not prolong survival.

The correlation between response rates and survival has been examined by reviewing 79 comparisons between arms with unequal response rates in 50 published trials of chemotherapy in advanced breast cancer. In 73% of comparisons the group with the higher response rate also demonstrated the longer median survival ($P<0.001$). Weighted linear regression showed a statistically significant relationship between relative response rates and survival ($P<0.001$). The number of patients in a comparison did not influence this relationship.

For the woman with metastatic breast cancer, systemic therapy with cytotoxic drugs is generally considered to be the only alternative to pure symptom relief after the failure of endocrine therapy. Whilst the chances of a long term cure for an individual are accepted to be remote, cytotoxics are used in the hope of both palliating symptoms and improving survival. The benefits of chemotherapeutic regimens must be weighed against their numerous and severe side effects.

The relative merits of cytotoxic regimens in controlling the disease are usually measured using the objective response rate. Such trials rarely demonstrate a statistically significant survival advantage in favour of the arm with the higher response rate. This could be because increased objective tumour response does not translate into a survival benefit. Another possible explanation is that individual trials lack sufficient statistical power to detect survival differences because such differences are small. In order to determine survival benefit we have therefore examined the correlation between response rates and survival by reviewing randomised trials in advanced breast cancer.

The relationship between response rate and survival was studied by comparing arms from the same trial. Arms from different trials were not compared directly because differing patient characteristics between trials would confound such comparisons that are normally compensated for by randomisation within a trial. The hypothesis tested was that if cytotoxic chemotherapy improved survival in advanced breast cancer there would be a tendency for the arm demonstrating the higher response rate to also demonstrate a longer median survival. The relationship between the odds ratio summarising the response rates in the two arms and the estimated hazard ratio calculated from the ratio of the median survivals was investigated.

### Materials and methods

This study uses only published data and papers; these will have been subject to peer review. Such an approach has been advocated by Chalmers *et al* (1986) to ensure data quality in meta-analyses.

Trials of chemotherapy in advanced breast cancer reporting survival data have been identified by reference to a comprehensive review article, (Macauley & Smith, 1986). Fifty of these trials had unequal response rates in two or more arms and could therefore be used to address the hypothesis tested in this study. Median trial size was 94 patients and ranged from 29 to 448 patients. There were a total of 6,056 patients in these trials. The inclusion of trials in the review article was not based on a desire to examine the relationship investigated in this study, hence selection bias, which may be a confounding factor in studies such as this (Chalmers *et al.*, 1986), has been reduced. For each trial the number of patients, the response rate and the median survival in each arm were recorded. A list of the trials considered may be obtained from the authors.

The data given for four trials were incomplete. In two trials, with a total of 93 patients, the exact median survival was not known in the arm with the higher response rate. The median survival was only known to exceed a given value which was longer than the median survival in the arm with the lower response rate. In both these cases this minimum value was used as the median survival, thus the survival benefit was underestimated in these two instances. In the other two trials, each of which had two arms, the numbers of patients in individual arms was not given, only the overall number of patients in the trial was supplied. In these cases equal numbers of patients were assumed to have been randomised to each arm. These two trials had a total of 173 patients.

Trials with more than two arms contributed more than one comparison to the study, e.g., trials with three arms contributed three comparisons corresponding to the three possible pairs formed from the arms. A comparison between two arms of a trial was considered to be a chemotherapy comparison if:

(1) the only difference between the regimens given to the two arms was one or more chemotherapeutic agents. Prednisone was not considered to be a chemotherapeutic agent. However, if two arms differed by one or more chemotherapeutic agents and prednisone the comparison was included in the study (this applied to 8 comparisons).

(2) if two arms differed only in the dose or schedule of one or more chemotherapeutic agents.

Data was collected in the following form:

| Arm | Number of responders | Number of nonresponders |
|---|---|---|
| A | $R_A$ | $N_A$ |
| B | $R_B$ | $N_B$ |

Each arm of the trial was individually coded and entered together with the response rate and median survival into a database created specifically for this purpose on a Prime 2655 computer. Statistical analysis was performed using BMDP Statistical Software, Programme version April 1985.

### *Response*

An estimate of the log odds ratio was used to summarise the

Correspondence: R.P. A'Hern.
*Present address: Department of Computing, Royal Marsden Hospital, Fulham Road, London SW3 6JJ, UK.
Received 8 September 1987; and in revised form, 8 March 1988.

relative response rates of each comparison. The rationale for the choice of this statistic is given in **Appendix I.** The convention was adopted of expressing the odds ratio as the odds of the group with the highest response rate divided by the odds of the group with the lower response rate. The natural logarithm of the odds ratio (LOR) was used in this study, i.e., they were taken to the base $e = 2.718$. The LOR is zero when the response rates are equal, because the OR was expressed as the odds of the arm with the higher response rate divided by the odds of the arm with the lower response rate, LOR is always greater than zero.

The formula used to calculate the odds ratio was:

$$\text{Odds ratio} = \frac{(R_A + 0.5)(N_B + 0.5)}{(N_A + 0.5)(R_B + 0.5)}$$

### *Survival*

The median survival is commonly used to summarise survival. The ratio of median survivals was chosen as the most appropriate method of summarising the relative survival in two arms in this study. This measure has the advantage of being an estimate of the hazard ratio if survival follows an exponential (constant hazard) model. A common assumption made when fitting such models to survival data is that the log hazard ratio is normally distributed, this makes the log hazard ratio a suitable dependent variable for linear regression. The hazard ratio was estimated by dividing the median survival of the group with the higher response rate by the median survival of the group with a lower response rate.

$$\frac{\text{Median survival arm A}}{\text{Median survival arm B}} = \text{Estimated Hazard Ratio (EHR).}$$

The natural logarithm of the estimated hazard ratio (LEHR) was employed. When the median survivals are identical LEHR is 0. LEHR is positive when the group with the improved response rate also has a longer median survival and negative when this group has a shorter survival. In one trial the median survivals were not given but the proportions surviving at a given time were supplied, the hazard ratio was estimated from these assuming an exponential survival model was applicable.

### *Methodology*

The correlations between LEHR and the number of patients in a comparison and LOR and the number of patients in a comparison, were both examined using Spearman's rank correlation.

The relationship between LOR and LEHR was investigated using weighted linear regression. The inverse of the variance of the log odds ratio was used as the weighting factor, thus greater weight was given to those comparisons in which the log odds ratio was known with greater precision, i.e., the comparisons with the most patients.

This weighting factor was therefore:

$$\frac{1}{\text{Variance}} = \frac{1}{\frac{1}{R_A+0.5} + \frac{1}{N_A+0.5} + \frac{1}{R_B+0.5} + \frac{1}{N_B+0.5}}$$

A further weighting factor was also included to compensate for the fact that the comparisons are not independent in trials with more than two arms. In a three arm trial, for example, there are two independent comparisons. In this instance each comparison was weighted by a factor of 2/3, the weight being the number of independent comparisons divided by the total number of comparisons. Similarly in a four arm trial a weighting factor of 3/6 was used.

The regression relationship was constrained so that LEHR was zero when the LOR was zero, i.e., if there is no difference in response rates there can be no difference in survival benefiting the group with the higher response rate.

The regression equation

$$\text{LEHR} = (A + B\ [\text{no. of pts}]) \times \text{LOR} + (C + D\ [\text{no. of pts}]) \times \text{LOR}^2$$

was fitted to the data in a stepwise fashion, where $A$, $B$, $C$ and $D$ are the regression coefficients.

Coefficients $A$ and $C$ represent the relationship between the LEHR and LOR, if they are zero this would indicate there is no evidence that a relationship exists.

Coefficients $B$ and $D$ allow for modification of the relationship between LEHR and LOR according to the number of patients in a comparison, these coefficients were used to assess bias, if they were zero it would indicate that the estimated relationship is independent of comparison size.

This method was extended to consider terms in LOR cubed.

### Results

There were a total of 79 comparisons between arms from the 50 trials reviewed.

The Spearman rank corrrelation between LOR and the total number of patients in a comparison was $-0.42$, ($P < 0.001$), hence a relationship exists between the relative response rates and the number of patients in a comparison.

The correlation between LEHR and the number of patients in a comparison was $-0.10$, (ns), suggesting that there is no relationship between the number of patients and survival difference.

Figure 1 shows a plot of LEHR *versus* LOR. If there were no association between survival (LEHR) and response (LOR) one would expect 50% of comparisons to show a favourable LEHR associated with the group with the highest response rate. In 73% of comparisons the arm with the higher response rate also demonstrated a longer median survival.

Further examination of this relationship was performed using weighted linear regression. The regression equation which gave the best fit to the data was found to be of the form

$$\text{LEHR} = A \times \text{LOR} + C \times \text{LOR}^2.$$

**Figure 1** A plot of the log estimated hazard ratio (LEHR) against the log odds ratio (LOR). In all of those comparisons with an LEHR greater than zero the longer median survival is in the group with the higher response rate. Comparisons with an LEHR less than zero showed a shorter median survival in the group with higher response.

Estimates of the coefficients $A$ and $C$ are shown in the following table. This equation was not improved by the inclusion of either term involving the number of patients in a comparison, or by a cubic term in LOR.

| | Coefficient | s.e. | $t$ value | $P$ value |
|---|---|---|---|---|
| $A$ | 0.380 | 0.07 | 5.14 | $P<0.001$ |
| $C$ | −0.116 | 0.05 | −2.28 | $P=0.02$ |

This corresponds to the equation:

$$\text{LEHR}=0.380\times\text{LOR}-0.116\times\text{LOR}^2.$$

The correlation coefficient $R$ between LEHR and LOR was 0.61, thus 37% of the variation seen in survival can be explained by variation in response rates. **Appendix II** contains statistical details of the fitting of the regression equation. Exclusion of the eight comparisons which included prednisone in one arm yielded similar results ($A=0.373$ and $C=-0.113$).

Table I shows hypothetical examples derived from the above equation. The expected improvement in median survival corresponding to differences in response rates between two arms of a randomised trial are shown. It has been assumed the arm with the lower response rate has a response rate of 20% and a mean survival of 18 months.

### Discussion

The strongest evidence for an effect of chemotherapy upon the survival of women with advanced breast cancer would come from a prospective randomised trial comparing an effective chemotherapeutic regimen against an arm receiving purely symptomatic treatment, i.e., a trial of a regimen with a high response rate *versus* an arm with a zero response rate. At present such a study would be unlikely to receive ethical committee approval. Indirect methods of addressing this question include the use of non-randomised series and the use of historical controls.

Powles *et al.* (1980) examined the notes of 78 patients who received no chemotherapy and 80 patients who had received one of three chemotherapeutic regimens. There was no survival difference between the two groups from the time of first detection of metastatic disease, in fact patients who had survived one year since the first detection of their metastasis appeared to do worse with chemotherapy. Survival was increased by a factor of four in the responders to cytotoxic agents.

This study generated a vitriolic correspondence. However its findings were to be duplicated by a similar retrospective review of 483 patients by Patel *et al.* (1986) who considered patients treated between 1942 and 1975. It was found that despite a changing trend in therapy away from radiation and additive hormones towards chemotherapy and ablative hormones there was no increase in survival time from first diagnosis, or metastatic survival time, (from appearance of first metastasis). Whilst a similar conclusion was drawn by Paterson *et al.* (1982), closer inspection of their data suggests that an improvement in survival achieved by combination chemotherapy when compared to historical controls may have been missed due to a type II error.

Postulating that 'some of the enthusiasm for combination chemothrapy regimes may be due to the premature reporting of protocol results or emphasis on response rates rather than survival', Ross *et al.* (1985) reviewed patients treated over three decades. During the 1950s most patients had received endocrine therapy with the emphasis shifting slightly towards the hesistant use of single agent chemotherapy in the 1960s, followed by a large swing during the 1970s, when the majority received combination cytotoxics. The retrospective analysis of survival even when corrected for confounding variables showed an improvement in the 1970s which was attributed to the change in treatment.

Attention has recently turned to the outcome of treatment by comparison of survival times of patients in randomised trials of various treatments (Brambilla *et al.*, 1976; Rubens *et al.*, 1977; Priestman *et al.*, 1978). A common feature in these trials is the improved survival of the responders when compared with the non-responders to the treatment being studied. Whilst studies like these show a benefit for responders they do not address the question as to whether or not the therapy under question is improving survival for the group as a whole.

The present study suggests that there is a positive correlation between improved response and improved survival though it is impossible to rule out the existence of publication bias. The relationship between relative response rates and relative median survivals suggests that for differences in response rates such as are commonly seen in trials of chemotherapy regimens only modest benefits in median survival would be expected. For example, a trial comparing two arms, one of which has a response rate of 40% and the other a response rate of 20%, would be expected to show a 30 % increase in median survival in the arm with the higher response rate.

If, for example, the arm with the 20% response rate had a median survival of 18 months one would expect the other arm to have a median survival of 23 months.

There are two possible criticisms of this study. Firstly, the median survivals only represent the survival experience up to the median survival time, so inferences cannot be drawn about survival beyond this time. Secondly, patients have to live long enough to experience a response, if one group has inferior survival due to chance it may also show a poorer response rate because some patients did not survive long enough to demonstrate a response. This effect would tend to enhance the relationship between response rates and survival.

The correlation between LOR and the number of patients in a comparison suggests the existence of publication bias, small trials may be more likely to be published if there is a large difference in response rates. However, there are other

**Table I** Hypothetical examples derived from the fitted regression equation. The expected improvement in median survival corresponding to differences in response rates between two arms of a randomised trial are shown. It has been assumed the arm with the lower response rate has a response rate of 20% and a median survival of 18 months.

| *Response rate in arm with higher response rate (%)* | *Estimated median survival (months)* |
|---|---|
| Baseline 20 | 18.0 |
| 30 | 21.4 |
| 40 | 23.4 |
| 50 | 24.4 |
| 60 | 24.5 |
| 70 | 23.6 |

**Table II** Predicted values of LEHR for given values of LOR over the range zero to 2.5. Predicted values for models without a quadratic term and with a quadratic term are shown

| | *Predicted LEHR* | |
|---|---|---|
| *LOR* | *Model* $A*LOR$ | *Model* $A*LOR+C*LOR^2$ |
| 0.0 | 0.0 | 0.0 |
| 0.5 | 0.12 | 0.16 |
| 1.0 | 0.23 | 0.26 |
| 1.5 | 0.35 | 0.31 |
| 2.0 | 0.47 | 0.30 |
| 2.5 | 0.59 | 0.23 |

factors which may cause or contribute to such a relationship. Firstly, such an association is a mathematical necessity. Suppose, for example, there were a number of comparisons looking at the same treatments which have given 'true' LOR. In small comparisons there will be a larger distribution of values estimating this value (due to a larger sampling error) than in large trials, if any comparisons gave an estimate falling below zero the odds ratio will then be reversed (since the group with the higher response rate has now changed). In large trials there will tend to be fewer high estimates of the LOR and also fewer falling below zero. Hence the average LOR will tend to be higher in the smaller trials. The second factor which may contribute to the relationship between LOR and the number of patients in a comparison is that trials which show a large difference in response rates may have fewer patients because they were terminated sooner having satisfied stopping criteria or exhausted enthusiasm for participation.

An important question not addressed by this study is the effectiveness of particular regimens in producing response and increasing survival, only an 'average' relationship between response and survival has been calculated. Thus it is possible that some regimens show no relationship, or a negative relationship and that others show a strong association. The inadequate size of many trials means that it may not be possible to assess the effect of many regimens on survival. Overviews employing individual patient data would maximise the use of the available information and may therefore be a worthwhile undertaking for those comparisons with large enough numbers of patients.

### Appendix I

Use of the log odds ratio to measure the association between treatment and response.

The log odds ratio was chosen as a measure of the association between treatment and response both because it is suggested by the logistic model and it remains valid under other models, see for example Cox, 1970.

The response rate in the arm of a trial depends on both the prognosis of the patients and the regimen they received. In terms of a logistic model the response rate $R_A$ can be written

$$R_A = \frac{1}{1+e^{-(cx+a)}}$$

where $x$ is a covariate measuring prognosis, the parameter $c$ measures the relationship between response rate and prognosis and $e=2.718$ is the base of natural logarithms. The parameter $a$ measures the dependence of the response rate on regimen A. Similarly, for regimen B, the response rate can be written

$$R_B = \frac{1}{1+e^{-(cx+b)}}$$

The odds of response given A is then

$$\frac{R_A}{1-R_A} = e^{cx+a} \quad (1)$$

and similarly for regimen B the odds of response are

$$e^{cx+b} \quad (2)$$

Dividing (1) by (2) and taking logs then gives the log odds ratio $a-b$. The effect of the regimens is thus reflected only in the magnitude of $a-b$ and is independent of prognosis. It would therefore be anticipated that comparisons of the same regimens, but from different trials, would have the same log odds ratio despite any differences in patient prognosis between trials. This statistic is thus suitable for comparisons across trials. The estimate of the log odds ratio recommended by Gart (1966) has been used here, this estimate was found to be preferable to other estimates by this author and has the advantage of being defined if the number of responders or non-responders in either of the two groups is zero. The high values of LOR seen in this study arise from such comparisons, or from comparisons with a low (<5%) response rate.

### Appendix II

The regression equation implies that for values of LOR in excess of 3.24 there would be poorer survival in the arm with the higher response rate. However, this conclusion should be regarded with caution in view of the sensitivity of fitted regression lines to outliers. Comparisons with high log odds ratios had a zero or low response rate in one arm. Though the model which gives the best fit to all the comparisons includes a quadratic term this term is no longer statistically significant if the comparisons with a zero response rate in one arm are excluded. It is worth noting in this context that the fitted values for the two models fitting a straight line and a curved line to all the comparisons are similar over the range to zero to 1.5 (90% or values of LOR fall within this range). The fitted values over the range 0 to 2.5 are shown in Table II.

### References

BRAMBILLA, C., DE LENA, M., ROSSI, A., VALAGUSSA, P. & BONNADONNA, G. (1976). Response and survival in advanced breast cancer aftr two non cross resistant combinations. *Br. Med. J.*, **i**, 801.

BRINKLEY, D. & HAYBITTLE, J.L. (1959). Results of treatment of carcinoma of the breast. *Lancet*, **i**, 86.

CHALMERS, T.C., LEVIN, H., SACKS, H.S. & 3 others (1987). Meta-analysis of clinical trials as a scientific discipline. I: Control of bias and comparison with large co-operative trials. *Statistics in Medicine 1986*, **6**, 315.

COX, D.R. (1970). *Analysis of Binary Data.* Methuen: London.

DEVITT, J.E. & ADVENT, D.A. (1977). Effects of current palliative treatment on the survival of patients with breast cancer. *Can. J. Surg.*, **20**, 46.

GART, J.J.(1966). Alternative analyses of contingency tables. *J. R. Stat. Soc., Ser. B*, **34**, 441.

LANGLANDS, A.O., POCKOCK, S.J., KERR, G.R. & GORE, S.M. (1979). Long term survival of patients with breast cancer: A study of the curability of the disease. *Br. Med. J.*, **ii**, 1247.

MACAULEY, V. & SMITH, I.E. (1986). Advanced breast cancer. In *Randomised Trials in Cancer: A Critical Review by Sites*, Slevin, M.L. & Staquet, M.J. (eds) p. 359. Raven: New York.

PATEL, J.K., NEMOTO, T., VEZERDIS, M., PETRELLI, N., SUH, O. & DAO, T.L. (1986). Does more intensive palliative treatment improve overall survival in metastatic breast cancer patients? *Cancer*, **57**, 567.

PATERSON, A.H.G., SZAFRAN, O., CORNISH, F., LEES, A.W. & HANSON, J. (1982). Effect of chemotherapy on survival in metastatic breast cancer. *Breast Cancer Res. Treat.*, **1**, 357.

POWLES, T.J., SMITH, I.E., FORD, H.T., COOMBES, R.C., JONES, J.M. & GAZET, J.-C. (1980). Failure of chemotherapy to prolong survival in a group of patients with metastatic breast cancer. *Lancet*, **i**, 580.

PRIESTMAN, T., BAUM, M., JONES, V. & FORBES, J. (1978). Treatment and survival in advanced breast cancer. *Br. Med. J.*, **ii**, 1673.

RUBENS, R.D., ARMITAGE, P., WINTER, P.J., TONG, D. & HAYWARD, J.L. (1977). Prognosis in inoperable stage III carcinoma of the breast. *Eur. J. Cancer*, **13**, 805.

ROSS, M.B., BUZDAR, A.U., SMITH, T.L. & 5 others (1985). Improved survival of patients with metastatic breast cancer receiving combination chemotherapy. *Cancer*, **55**, 341.